# Supplementary material for: MetaQTL: a package of new computational methods for the meta-analysis of QTL mapping experiments
Source: BMC Bioinformatics. 2007 Feb 8;8:49. doi: 10.1186/1471-2105-8-49 (PMC1808479; doi:10.1186/1471-2105-8-49)
Supplement: Additional File 7 — MetaQTL Package : jar file and tutorial. This Zip archive contains both the MetaQTL JAR file and the files of the tutorial. [file 1471-2105-8-49-S7.zip › org.inra.metaqtl/doc/org/metaqtl/bio/IBioOntology.html]

IBioOntology


|  |  |  |
| --- | --- | --- |
| **Overview** \| **Package** \| **Class** \| **Use** \| **Tree** \| **Deprecated** \| **Index** \| **Help** | |  |
| **PREV CLASS**   **NEXT CLASS** | **FRAMES**    **NO FRAMES**     **All Classes** |  |
| SUMMARY: NESTED \| FIELD \| CONSTR \| METHOD | DETAIL: FIELD \| CONSTR \| METHOD |  |


---


## org.metaqtl.bio Interface IBioOntology

**All Superinterfaces:**: IBioAdaptable, IBioEntity

**All Known Implementing Classes:**: Ontology

---

``` public interface IBioOntology extends IBioEntity ```

This interface defines some rules to handle a ontology.
Here we assume that the onotlogy can be represented by a simple
directed acyclic graph, i.e a parent-child linking between the
concepts (if A is child of B then B can't be a child of A).

---

| **Method Summary** | |
| --- | --- |
| `java.lang.String` | `getFunction()`             Returns the function of the ontology |
| `IBioOntologyTerm` | `getRoot()`             Returns the root of the ontology tree. |
| `IBioOntologyTerm` | `getTerm(IBioOntologyTerm term)`             Returns if exists the term in the ontology which matches the given one. |
| `IBioOntologyTerm` | `getTerm(java.lang.String termName)`             This method looks into the ontology if there is a term with the given name and returns it. |
| `void` | `setRoot(IBioOntologyTerm root)`             Sets the root of the ontology. |

**Methods inherited from interface org.metaqtl.bio.IBioEntity**

`getName, getParent, getProperties, getType, setName, setProperties`

**Methods inherited from interface org.metaqtl.bio.IBioAdaptable**

`getBioAdapter`

**Method Detail**

### getFunction

```
java.lang.String getFunction()
```

:   Returns the function of the ontology

    :   **Returns:**

---


### getRoot

```
IBioOntologyTerm getRoot()
```

:   Returns the root of the ontology tree.

    :   **Returns:**: the root of the ontology tree.

---


### setRoot

```
void setRoot(IBioOntologyTerm root)
```

:   Sets the root of the ontology.

    :   **Parameters:**: `root` - the root of the ontology

---


### getTerm

```
IBioOntologyTerm getTerm(IBioOntologyTerm term)
```

:   Returns if exists the term in the ontology which
    matches the given one.

    :   **Parameters:**: `term` - the term to look for in the ontology. **Returns:**: the term if found, null otherwise.

---


### getTerm

```
IBioOntologyTerm getTerm(java.lang.String termName)
```

:   This method looks into the ontology if there is
    a term with the given name and returns it.

    :   **Parameters:**: `termName` - the name of the term to find. **Returns:**: the term or null if not found.


---


|  |  |  |
| --- | --- | --- |
| **Overview** \| **Package** \| **Class** \| **Use** \| **Tree** \| **Deprecated** \| **Index** \| **Help** | |  |
| **PREV CLASS**   **NEXT CLASS** | **FRAMES**    **NO FRAMES**     **All Classes** |  |
| SUMMARY: NESTED \| FIELD \| CONSTR \| METHOD | DETAIL: FIELD \| CONSTR \| METHOD |  |


---
